# Undetected dysglycaemia common in primary care patients treated for hypertension and/or dyslipidaemia: on the need for a screening strategy in clinical practice. A report from EUROASPIRE IV a registry from the EuroObservational Research Programme of the European Society of Cardiology

**DOI:** 10.1186/s12933-018-0665-4

**Published:** 2018-01-24

**Authors:** Bahira Shahim, Viveca Gyberg, Dirk De Bacquer, Kornelia Kotseva, Guy De Backer, Oliver Schnell, Jaakko Tuomilehto, David Wood, Lars Rydén

**Affiliations:** 1 Cardiology Unit, Department of Medicine, Heart and Vascular Theme, Karolinska Institute, Karolinska University Hospital, 171 76 Stockholm, Sweden; 2 Centre for Family Medicine, Department of Neurobiology, Care Sciences and Society, Karolinska Institute, Huddinge, Stockholm, Sweden; 3 Department of Public Health, Ghent University, Ghent, Belgium; 4 Department of Cardiovascular Medicine, National Heart and Lung Institute, Imperial College London, London, UK; 5 Forschergruppe Diabetes e.V. at the Helmholtz Center, Munich, Germany; 6 Department of Neurosciences and Preventive Medicine, Danube-University Krems, Krems, Austria; 7 Chronic Disease Prevention Unit, National Institute for Health and Welfare, Helsinki, Finland; 8 Diabetes Research Group, King Abdulaziz University, Jeddah, Saudi Arabia; 9 Dasman Diabetes Institute, Dasman, Kuwait City, Kuwait

**Keywords:** Type 2 diabetes, Impaired glucose tolerance, Screening, FINDRISC, Dyslipidaemia, Hypertension, Primary care

## Abstract

**Background:**

Dysglycaemia defined as type 2 diabetes (T2DM) and impaired glucose tolerance (IGT), increases the risk of cardiovascular disease (CVD). The negative impact is more apparent in the presence of hypertension and/or dyslipidaemia. Thus, it seems reasonable to screen for dysglycaemia in patients treated for hypertension and/or dyslipidaemia. A simple screening algorithm would enhance the adoption of such strategy in clinical practice.

**Objectives:**

To test the hypotheses (1) that dysglycaemia is common in patients with hypertension and/or dyslipidaemia and (2) that initial screening with the Finnish Diabetes Risk Score (FINDRISC) will decrease the need for laboratory based tests.

**Methods:**

2395 patients (age 18–80 years) without (i) a history of CVD or TDM2, (ii) prescribed blood pressure and/or lipid lowering drugs answered the FINDRISC questionnaire and had an oral glucose tolerance test (OGTT) and HbA1c measured.

**Results:**

According to the OGTT 934 (39%) had previously undetected dysglycaemia (T2DM 19%, IGT 20%). Of patients, who according to FINDRISC had a low, moderate or slightly elevated risk 20, 34 and 41% and of those in the high and very high-risk category 49 and 71% had IGT or T2DM respectively. The OGTT identified 92% of patients with T2DM, FPG + HbA1c 90%, FPG 80%, 2hPG 29% and HbA1c 22%.

**Conclusions:**

(1) The prevalence of dysglycaemia was high in patients treated for hypertension and/or dyslipidaemia. (2) Due to the high proportion of dysglycaemia in patients with low to moderate FINDRISC risk scores its initial use did not decrease the need for subsequent glucose tests. (3) FPG was the best test for detecting T2DM. Its isolated use is limited by the inability to disclose IGT. A pragmatic strategy, decreasing the demand for an OGTT, would be to screen all patients with FPG followed by OGTT in patients with IFG.

## Introduction

Dysglycaemia, defined as type 2 diabetes (T2DM) or impaired glucose tolerance (IGT), is an important risk factor for cardiovascular disease (CVD), the commonest cause of death globally [1]. Screening for dysglycaemia is performed by means of three blood tests: glycated haemoglobin A1c (HbA1c), fasting plasma glucose (FPG) and a 2 hour post load plasma glucose (2hPG), the latter only obtainable by means of an oral glucose tolerance test (OGTT) [2]. Several scoring systems for estimating the future risk of T2DM have been developed. One of them, the FINDRISC questionnaire, has been validated in several European populations. FINDRISC classifies the respondent as having a low, moderate, high or very high risk of developing T2DM during the forthcoming decade. If an individual is at a high or very high risk, an OGTT is recommended for further investigation [3].

Importantly, not only T2DM but also its preceding, hyperglycaemic state IGT increases the risk of CVD [4, 5]. Other intermediate hyperglycaemic states include impaired fasting glucose (IFG) and “high-risk” glycated haemoglobin A1c (HbA1c). The Atherosclerosis Risk in Communities (ARIC) study reported that people with “high risk” HbA1c are at increased risk of developing diabetes and CVD independent of FPG [6], but a postload glucose was not included in this study. Other population based studies among them the Diabetes Epidemiology: Collaborative Analysis of Diagnostic Criteria in Europe (DECODE), a substudy of the DECODE population and the Framingham Offspring Study demonstrated that the 2hPG is a significantly stronger predictor of future CVD and all-cause mortality than FPG and HbA1c [7–9]. Similar findings were recently presented from a large European population of patients with coronary artery disease [10].

Screening for dysglycaemia is encouraged by the fact that T2DM can be prevented or delayed by approximately 50% with lifestyle and/or pharmacological interventions [11–13]. Although advocated by contemporary guidelines systematic screening of high risk people is infrequently implemented [2]. A concern is therefore that large proportions of dysglycaemic individuals remain unrecognized, thereby deprived preventive opportunities.

This study tested the hypotheses that (1) if appropriately screened the prevalence of dysglycaemia is high in patients free from CVD but treated for hypertension and/or dyslipidaemia, and (2) that initiating screening with the FINDRISC questionnaire would limit the need for more expensive and time consuming blood tests. These hypotheses were tested within the auspices of the European Action on Secondary Prevention through Intervention to Reduce Events-IV (EUROASPIRE-IV), a large cross-sectional survey.

## Materials and methods

### Study population

The primary care arm of EUROASPIRE IV was carried out in 14 European countries January 2014–April 2015 (Bosnia and Herzegovina, Bulgaria, Croatia, Kazakhstan, Lithuania, Poland, Portugal, Romania, Russian Federation, Serbia, Spain, Sweden, Ukraine and the UK). Within each country one or more geographical areas with a defined population were selected. A sample of general practices serving that population was identified according to the structure of the local health services.

**Fig. 1:**
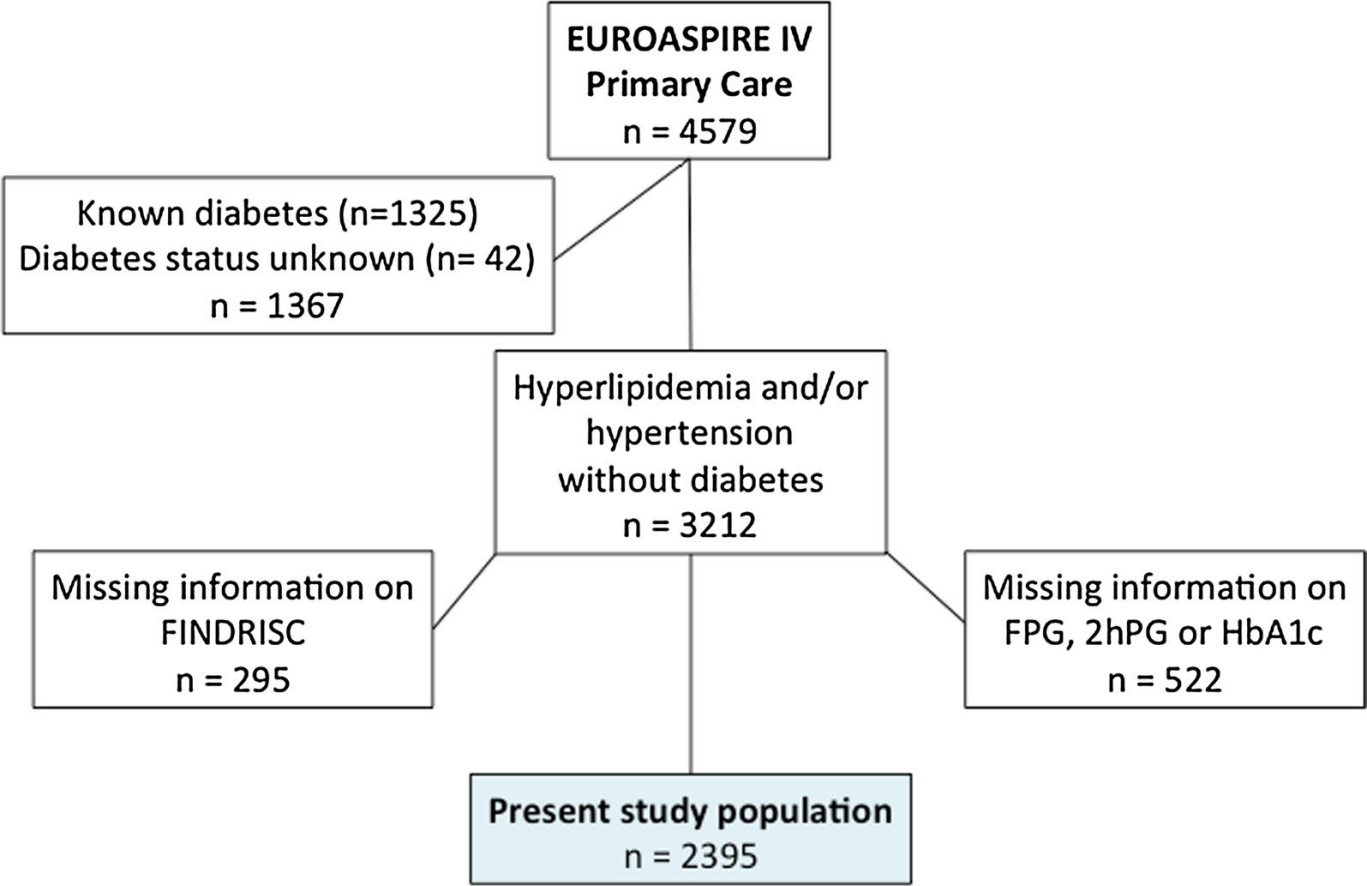
Patient flow chart

Within each general practice men and women ≥ 18 to < 80 years at the time of identification, without a history of CVD, coronary or other atherosclerotic disease, who had been prescribed one or more of the following treatments: (i) blood pressure lowering drugs and/or (ii) lipid lowering drugs and/or (iii) glucose lowering (diet and/or oral drugs and/or insulin) since ≥ 6 months to < 3 years prior to the date of interview, were retrospectively identified from practice records. The total number of such patients was 4579 of whom 1325 with a history of treatment for diabetes and 42 who lacked information on diabetes were excluded. Out of the remaining 3212 patients the information on plasma glucose or HbA1c (n = 522) and the FINDRISC questionnaire (n = 295) was incomplete leaving 2395 as the study population (Fig. 1).

## Methods

Centrally trained research staff undertook data collection using standardised methods and the same instruments in all centres. They reviewed patient medical records and interviewed and examined the patients at the general practice or home at least 6 months after the prescription of blood pressure, lipid or glucose lowering therapy. A detailed description of this procedure and applied definitions have been presented elsewhere [14]. Methods of particular relevance for the present investigation are described below.

*Height* (kg) and *weight* (cm) were recorded in light indoor clothes without shoes (Scales 701 and Measuring stick model 220; SECA Medical Measuring Systems and Scales, Birmingham, UK). *Waist circumference* (cm) [15] was measured using a metal tape applied horizontally at the point midway in the mid-axillary line between the lowest rim of the rib cage and the tip of the hip bone (superior iliac crest) with the patient standing.

*Blood pressure* was measured twice on the right upper arm in a sitting position using an automatic digital sphygmomanometer (Omron M6; OMRON Corporation, Kyoto, Japan) and the mean was used for the analyses.

*Physical activity* was assessed with the following question: “Which of the following four alternatives describes your level of activity outside work in the best way?” (a) No physical activity weekly, (b) only light physical activity in most weeks, (c) vigorous physical activity at least 20 min once or twice a week (d) vigorous physical activity for at least 20 min three or more times a week.

The *FINDRISC* [16] was integrated as a part of the interview conducted by the research staff. It includes eight questions/items (age, BMI, waist circumference, intake of fruit and vegetables, physical activity, high blood pressure, history of high glucose value and family history of diabetes). Depending on the response to each question a score is set, the sum of which allocates the responding person to one of the following categories: < 7 low risk implying that 1 in 100 will develop T2DM within the next decade; 7–11 slightly elevated risk, 1 in 25 will develop T2DM; 12–14 moderate risk, 1 in 6 will develop T2DM; 15–20 high risk, 1 in 3 will develop T2DM; and > 20 very high, 1 in 2 will develop T2DM within the next decade. Applying a threshold of ≥ 9 revealed a sensitivity of 78%, a specificity of 77% and a predictive value of a negative test of 99% [3].

### Laboratory investigations

Venous blood was drawn in the fasting state (≥ 10 h) into a tube containing clot activator (Venosafe, Terumo Europe, Leuven, Belgium) for lipid assays and into a potassium ethylenediaminetetraacetic acid (EDTA) tube for HbA1c assay. Total and high-density lipoprotein (HDL) cholesterol and triglycerides were analysed in serum and HbA1c in whole blood on a clinical chemistry analyzer (Abbot Architect analyzer; Abbott Laboratories, Abbott Park, Illinois, USA) at a central laboratory (Disease Risk Unit, National Institute for Health and Welfare, Helsinki, Finland) accredited by Finnish Accreditation Service fulfilling the requirements of the standard SFS-EN ISO/IEC 17025:2005. Total serum cholesterol was measured using an enzymatic and high-density cholesterol (HDL-C) with a homogenous method for direct measurement while triglycerides were measured by an enzymatic glycerol phosphate oxidase method. Low-density lipoprotein (LDL-C) cholesterol was calculated according to Friedewald’s formula [17]. Glycated HbA1c was analysed with an immunoturbidimetric method and expressed in mmol/mol according to IFCC and % according to Diabetes Control and Complications Trial (DCCT). The glomerular filtration rate (eGFR) was estimated from serum creatinine by means of the Chronic Kidney Disease Epidemiology Collaboration equation (CKD-EPI) [18].

The OGTT was performed using 75 g of anhydrous glucose in 200 ml of water in the morning after at least 10 h of fasting. Blood for FPG was drawn before intake of the glucose with a dip safe from the EDTA-tube in which the HbA1c was collected. Samples for 2hPG were drawn from whole venous blood using an EDTA-tube. Plasma glucose was analysed locally with a photometric point-of-care technique (Glucose 201 + , HemoCue^®^, Ängelholm, Sweden). Regression analysis between the HemoCue^®^ instrument, and standard isotope dilution gas chromatography–mass spectrometry (IDGC–MS) showed a slope of 1.051 (95% confidence interval 1.031–1.071) an intercept of − 0.222 (95% CI − 0.016 to − 0.428; r = 0.994). The mean deviation was 0.24 mmol/l (2.0%). Values obtained with the HemoCue^®^ instrument were in 69% within 5%, in 91% within 10%, and always within 14.3% of the IDGC–MS method [19]. Since the HemoCue^®^ method is cholesterol sensitive due to the measurement in very small volumes with higher levels of glucose with low cholesterol the glucose values were corrected according to the formula: HemoCue^®^ glucose + 0.15 × (total cholesterol − 6). The values were converted from whole venous blood to plasma applying the formula by Carstensen et al. [19]: plasma glucose = 0.558 + 1.119 × whole blood glucose. Standardized use of the equipment was ascertained through central training of the data collectors, and retrieval of HemoCue^®^-cuvette storage information, and validation sheets from a selection of the participating centres.

### Definitions

*Hyperglycaemia* defined as IFG, IGT, “high risk HbA1c” or newly detected T2DM was diagnosed according to WHO and ADA [20, 21] as outlined in Table 1.

**Table 1:** Definitions of dysglycaemia according to WHO and ADA

Test/diagnose	Cut off level	
HbA1c	% DCCT	mmol/mol IFCC
High risk HbA1c	5.7–6.4	39–47
Diabetes	≥ 6.5	≥ 48

Plasma glucose	mmol/l	mg/dl
Impaired fasting glucose		
Fasting	6.1–6.9	110–125
2 h postload	< 11.0	< 200
Impaired glucose tolerance		
Fasting	< 7.0	< 126
2 h postload	≥ 7.8–11.0	≥ 140–199
Diabetes		
Fasting	> 7.0	> 126
2 h postload	≥ 11.1	≥ 200

*Elevated LDL*-*C* concentration was defined as a level ≥ 2.5 mmol/l.

*Normal kidney function* was defined as eGFR ≥ 60 ml/min/1.73 m^2^.

*Overweight* was defined as a body mass index (BMI) 25.0–29.9 kg/m^2^ and *obesity* as a BMI ≥ 30 kg/m^2^. *Central obesity* was defined as a waist circumference of ≥ 88 cm for women and ≥ 102 cm for men [15].

*Blood pressure* was defined as elevated if systolic blood pressure (SBP) was ≥ 140 mmHg and/or diastolic blood pressure (DBP) ≥ 90 mmHg.

*Smoking* was defined as self-reported smoking or an exhaled carbon monoxide > 10 ppm [22].

*The physical activity target* was defined as vigorous physical activity outside work for ≥ 20 min at least once/week.

*The educational level* was defined as low if only primary school or less had been completed.

### Data management

The EURObservational Research Programme at the European Heart House (Nice, France) was in charge of data management. All data were collected electronically through web-based data entry using a unique identification number for country, centre and individual. The data were submitted via the Internet to the data management centre where checks for completeness, internal consistency and accuracy were performed. All data were stored under the provisions of the National Data Protection Regulations.

### Statistical analyses

Continuous variables were summarised according to their mean and standard deviation (SD); dichotomous variables as percentages (n). For comparing characteristics of included and excluded patients p-values were obtained from linear and logistic regression models adjusted for age and gender. All statistical analyses were undertaken using SAS statistical software (release 9.4) at the Department of Public Health, Ghent University, Belgium.

## Results

### Clinical characteristics

Pertinent clinical characteristics of included (n = 2395) and excluded (n = 817) patients are presented in Table 2; those excluded from the analysis were more often smokers, less physically active and had a lower educational level, higher diastolic blood pressure, FPG and HbA1c and were more frequently using statins and calcium channel blockers. Among the included patients 94% had a normal kidney function and the eGFR was unrelated to FPG, 2 h-PG and HbA1c in all patients.

**Table 2:** Pertinent characteristics of included and excluded patients

Variable	Included (n = 2395)	Excluded (n = 817)	*p* value*
Age (years; mean ± SD)	58.1 (7.8)	57.9 (11.7)	0.71
Female gender (%, n)	60.8 (1457/2395)	57.8 (472/817)	0.13
BMI (kg/m^2^; mean ± SD)	29.4 (5.0)	29.3 (4.7)	0.38
Obesity (%, n)	40.8 (976/2395)	39.4 (318/808)	0.52
Central obesity (%, n)	60.7 (1452/2393)	60.6 (477/787)	0.81
Smoking			
Current (%, n)	16.0 (382/2395)	19.7 (161/817)	0.03
Past (%, n)	26.2 (628/2395)	24.8 (203/817)	0.27
Hypertension (%, n)	49.1 (1175/2392)	52.1 (420/806)	0.19
Blood pressure (mmHg)			
Systolic (mean ± SD)	138.6 (17.9)	139.6 (18.3)	0.20
Diastolic (mean ± SD)	82.8 (10.3)	84.5 (10.7)	0.0002
Blood lipids (mmol/l)			
Total cholesterol (mean ± SD)	5.63 (1.19)	5.60 (1.18)	0.63
HDL cholesterol (mean ± SD)	1.31 (0.32)	1.30 (0.34)	0.29
LDL cholesterol (mean ± SD)	3.58 (1.03)	3.54 (1.01)	0.52
Triglycerides (mean ± SD)	1.66 (1.02)	1.73 (1.19)	0.56
Plasma glucose (mmol/l)			
Fasting (mean ± SD)	6.24 (0.91)	6.40 (0.83)**	0.02
2 h postload (mean ± SD)	7.34 (2.28)	7.62 (2.26)***	0.13
HbA1c (%)(mean ± SD)	5.67 (0.50)	5.75 (0.67)****	0.0003
Pharmacological treatment			
ASA/antiplatelets (%, n)	27.8 (665/2391)	25.0 (204/815)	0.14
Lipid lowering (%, n)	31.0 (741/2391)	39.1 (318/814)	< 0.0001
Beta-blockers (%, n)	30.5 (730/2392)	31.5 (256/813)	0.53
ACE-inhibitors (%, n)	48.2 (1153/2392)	49.0 (399/814)	0.71
AT-II receptor blockers (%, n)	18.9 (452/2392)	19.8 (161/813)	0.56
Calcium channel blockers (%, n)	22.4 (535/2392)	26.3 (214/814)	0.03
Diuretics (%, n)	29.4 (703/2391)	31.5 (257/815)	0.21
Low educational level (%, n)	10.5 (250/2382)	14.7 (120/816)	0.002
Low or moderate physical activity (%, n)	55.4 (1295/2337)	61.5 (487/792)	0.001

Obesity-BMI ≥ 30 kg/m^2^

Central obesity-a waist circumference of ≥ 88 cm for women and ≥ 102 cm for men

Hypertension—systolic blood pressure (SBP) ≥ 140 mmHg and/or diastolic blood pressure (DBP) ≥ 90 mmHg

Smoking—self-reported smoking or an exhaled carbon monoxide > 10 ppm

The physical activity target was defined as vigorous physical activity outside work for ≥ 20 min at least once/week

Low educational level—if completed only primary school or less

*HDL*-*C* high-density lipoprotein cholesterol, *LDL*-*C* low-density lipoprotein cholesterol, *ACEI* angiotensin-converting enzyme inhibitor, *AT*-*II* angiotensin II receptor blockers, *BMI* body mass index

* Significance of the difference between groups, adjusted for age and gender; **N = 177; ****N = 167; ****N = 668

### Glycaemic status

**Fig. 2:**
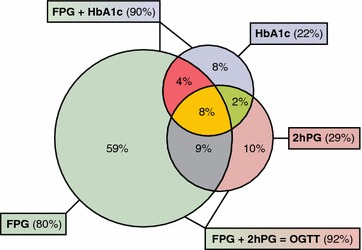
The 492 patients with newly detected T2DM. Proportions and their overlap between screening with FPG ≥ 7 mmol/l, 2hPG ≥ 11.1 mmol/l, HbA1c ≥ 6.5%/48 mmol/mol and combinations commonly used in clinical practice (FPG + HbA1c and FPG + 2 hPG)

The distribution of T2DM by diagnostic test is presented in Fig. 2. A FPG alone identified 80% of those with previously unknown T2DM while FPG in combination with 2hPG identified 92% and FPG together with HbA1c 90% of them.

A total of 455 patients (19%) were identified as having T2DM by the OGTT of whom 396 (87%) were detected by FPG alone. Including HbA1c ≥ 48 mmol/mol (DCCT ≥ 6.5%) as an additional diagnostic test the number of patients with newly detected T2DM increased by 13 to 468 (19.5%). Eight percent of the patients were detected by all three tests. Patients who were detected as having T2DM with HbA1c (8%) or 2hPG (10%) alone did not differ significantly from each other regarding age (mean ± SD = 59.0 ± 10.3 vs. 61.5 ± 11.9; p = 0.21), gender (males = 55.3% vs. 65.3%; p = 0.17) or FINDRISC score (mean ± SD 12.6 ± 3.5 vs. 13.9 ± 4.0; p = 0.38).

The 2hPG revealed that another 479 patients (20%) had IGT. The FPG classified 1495/2395 patients (62%) as having IFG (FPG = 5.6–6.9 mmol/l) including 395/479 of the patients with IGT. A “high risk” HbA1c [IFCC 39–47 mmol/mol (DCCT 5.7–6.4%)] was present in 72% of the total patient population.

Among the included patients 48.2–29.4% were treated with β-blockers or calcium channel blockers, respectively. The prevalence of newly detected dysglycaemia among patients on β-blockers was 41.0% (299/730) and among those without β-blockers 38.2% (634/1662) (p = 0.23 after adjustment for age and sex). About 43.4% (232/535) of the patients treated with calcium channel blockers had dysglycaemia compared to 37.8% (701/1857) in patients not treated with calcium channel blockers (p = 0.11 after adjustment for age and sex). In patients treated with statins 35.9% (256/713) had newly detected dysglycaemia compared to 40.4% (677/1678) among those who were not treated with statins (p = 0.0007 after adjustment for age and sex). Eighty-five percent of the patients were Caucasians. Among the remaining 15% 173 were Asian of whom 53 (30.6%) had newly detected T2DM. The distribution of dysglycaemia according to the different screening tests was similar to that in the Caucasian population.

### FINDRISC

**Fig. 3:**
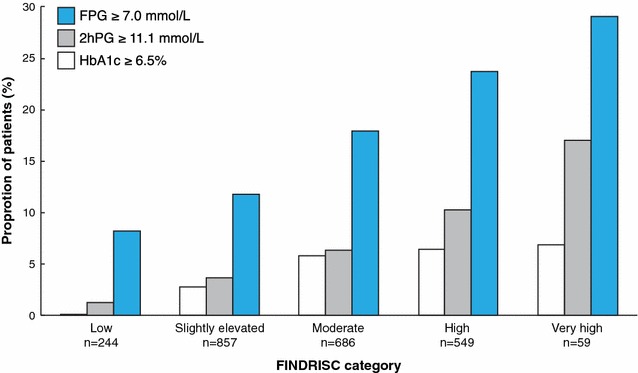
Proportion of patients with newly detected T2DM by either FPG, 2hPG or HbA1c in each of the FINDRISC categories. The total numbers of patients in each FINDRISC category are indicated below each bar

**Fig. 4:**
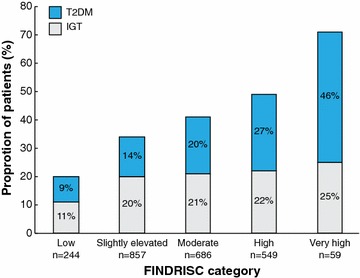
Proportion of patients with dysglycaemia according to the OGTT (FPG + 2hPG) in each of the FINDRISC categories. The total number of patients in each FINDRISC category are indicated below each bar

The distribution of patients according to FINDRISC was: low risk to develop diabetes 10% (n = 244), slightly elevated risk 36% (n = 857), moderate risk 29% (n = 686), high risk 23% (n = 549) and very high risk 3% (n = 59). The proportion of patients with T2DM detected by FPG, 2hPG and HbA1c in relation to their FINDRISC category increased linearly (Fig. 3). The relationship between FINDRISC category and dysglycaemia (T2DM and IGT combined) diagnosed by FPG and 2hPG is shown in Fig. 4. Of patients, who according to FINDRISC had a low, moderate/slightly elevated risk 20, 34 and 41% had IGT or T2DM respectively. The corresponding proportions for the high and very high-risk category were 49 and 71%.

## Discussion

The main findings in this investigation of patients free from CVD but on treatment for hypertension and/or dyslipidaemia were that: (1) the prevalence of previously undetected dysglycaemia was very high; (2) a large proportion of the present patient population in the lower FINDRISC categories for T2DM had dysglycaemia; (3) even if a FPG was the best single test for detecting T2DM it left a considerable proportion of patients with dysglycaemia undetected.

### Dysglycaemia in the presence of other risk factors for CVD

The present data from a European primary care population consolidate that dysglycaemia is a very common and global condition in patients with one or two other risk factors for CVD. The prevalence of screen detected dysglycaemia was 37% (IGT 11%; T2DM 17%) in the Impaired Glucose Tolerance and Long-Term Outcomes Observational (IGLOO) study, based on 1377 Mediterranean participants without known CVD but with one or more cardiovascular risk factors [23]. A similar proportion, 41%, was reported by the ARIC study [24] screening 8286 middle-aged American participants with the OGTT, of whom many with hypertension, dyslipidaemia and/or central obesity. Further, 14% had screen detected T2DM in a cross-sectional population-based study from Kuwait in which more than half of the participants were overweight or obese but without any history of CVD [25].

In the Interventions in Europe and Worldwide (PREVIEW) among 424 New Zealanders with overweight and FINDRISC score of ≥ 12, about 65% had undetected prediabetes and 7% T2DM when screened with the OGTT [26]. A higher FINDRISC score was significantly associated with prediabetes and T2DM (p = 0.02). The prevalence of dysglycaemia among those with lower FINDRISC score was, however, not investigated.

### The importance of dysglycaemia screening

It has since long been established that dysglycaemia is an important risk factor for future CVD [2]. Other important risk factors for CVD are dyslipidaemia and hypertension in the presence of which dysglycaemia causes a dramatic increase in the risk for future CVD [27, 28]. The outcome of dysglycaemia screening is therefore a prerequisite for the institution of a multifactorial risk factor management as strongly emphasized by contemporary guidelines for cardiovascular disease prevention [2, 29]. It may be argued that patients under treatment for hypertension and/or hyperlipidaemia should have received life-style advice, the corner-stone in dysglycaemia management. Still 40% had undetected T2DM or IGT indicating a need for intensified life-style advice most likely supplemented by glucose lowering drugs in patients with newly detected T2DM. As regards IGT the experiences from the Da Qing study (11) and the Finnish diabetes prevention study [30] demonstrate that an improved life-style significantly reduced the development of overt T2DM during prolonged periods of follow up. Moreover, micro- and macro-vascular complications including cardiovascular mortality was reduced in the Da Qing study.

Opportunistic screening for dysglycaemia, as applied in the present study, is not contradicted by the ongoing debate on the value of population based screening for diabetes based on the inconclusive results of The Anglo–Danish–Dutch Study of Intensive Treatment in People with Screen-Detected Diabetes in Primary Care (ADDITION) [31–34]. A reasonable assumption is that ADDITION, testing a community based screening programme in a general population, does not reflect the benefit of screening for dysglycaemia in a population at higher risk due to already diagnosed risk factors. In such population, an early instituted and comprehensive treatment has a more obvious rationale. A remaining question is how to screen. The willingness is by apparent reasons dependent of screening tools that are easy to handle and affordable. The simplicity of the FINDRISC questionnaire makes it easy to apply for the initiation of primary prevention in high risk people [13, 35]. In addition it is valuable for the detection of IGT, relates to markers of insulin resistance [36] and predicts CVD events and mortality [37].

### FINDRISC in relation to blood glucose measurements

In the present investigation it was hypothesized that the use of FINDRISC as a first step in dysglycaemia screening would reduce the need for blood tests. This assumption could not be verified since a large proportion (20–40%) of the participants were dysglycaemic already in the low-moderate FINDRISC risk categories. The most reasonable explanation is the high overall prevalence of dysglycaemia in the present population of people treated for—hypertension and/or dyslipidaemia. FINDRISC was based on a sample derived from the general population and its discriminatory ability is reported to be less in populations at higher risk [38]. Furthermore, the different FINDRISC components may differ by race and gender as reported by a subset of the ARIC study [39]. For example, waist circumference was more predictive of incident diabetes than BMI among black men but not in white women. An alternative to the FINDRISC for the prediction of dysglycaemia may to estimate lipoprotein (a), triglyceride/HDL-C ratio and triglyceride glucose index, which has been associated with insulin resistance and the risk of future incident diabetes [40–42].

Several studies added biochemical measures, in particular FPG to improve the predictive capacity of risk scoring models [43]. Thus, screening was based on the IFG cut point (≥ 6.1 mmol/l) in the ARIC study to be followed by a clinical detection rule based on the risk factors of the metabolic syndrome and CVD for those below this value. With this strategy 86% of those with T2DM and 66% of all hyperglycaemia cases were detected, identifying 42% of the study population as screen positive [24]. In the IGLOO study, addressing opportunistic screening strategies based on the FINDRISC in individuals with one or more cardiovascular risk factors, the best screening strategy seemed to be based on FPG measurement in all participants and the performance of OGTT only in those with a FPG ≥ 5.6 mmol/l [23]. Based on this strategy, in which an OGTT was required for 56% of the studied population, 97% of the participants with T2DM and 78% of those with IGT was identified.

In the present study, the combination of FPG and 2hPG identified 92% of those with T2DM and the 2hPG another 20% with IGT. The superiority of this combination corresponds to the outcome of a previous study from EUROASPIRE IV on glycaemic screening of patients with established coronary artery disease [44] and in a recent population based study population consisting of overweight and obese subjects free from cardiovascular disease [45]. “High risk” HbA1c [DCCT 5.7–6.4% (IFCC 39–47 mmol/mol)] classified a majority (72%) of the present population as having glucose perturbations. This proportion is reasonably too high. Furthermore “high risk” HbA1c is less sensitive than IFG and IGT to detect individuals with insulin resistance and ß-cell dysfunction [46]. According to a WHO expert group, “high risk” HbA1c is a less effective diagnostic tool than FPG and 2hPG and current evidence is insufficient to make any formal recommendation on the interpretation of HbA1c levels < 6.5% [20, 47].

An alternative, limiting OGTT to 62% of the population (those with IFG), would be to start screening with a FPG continuing with an OGTT only in patients not fulfilling the criteria for T2DM. With such strategy 18% of patients with IGT would be missed. It has to be debated whether this is acceptable.

### Strengths and limitations

A major strength of EUROASPIRE IV is that data are based on interviews and standardised examinations of a large cross-sectional European population of well-characterised high-risk patients treated for hypertension and/or dyslipidaemia. Thus, the studied population is representative for a common at-risk group which, so far free from CVD, should be of particular interest for targeted life-style based interventions [4, 5, 48]. All tests (FPG, 2hPG and HbA1c) were uniformly undertaken by centrally trained staff members using the same methodology including a central laboratory. A limitation is that, as in almost all similar studies, dysglycaemia was based on a single glucose recording rather than, as recommended by WHO for a clinical diagnosis, repeated measurements. Moreover, it may be argued that patients who, due to missing data, were excluded had a somewhat higher risk profile (Table 2). This may, if of any importance, contribute to an underestimation of the true prevalence of dysglycaemia. Markers of dysglycaemia may vary by race and ethnicity [49, 50]. The vast majority of the present population was of Caucasian origin limiting the generalisability of the present findings to such populations.

Statin use may increase the risk for T2DM as well as β-blockers and possibly calcium channel blockers [51]. In the present study patients on statins had a lower proportion of dysglycaemia and there were no significant differences for β-blockers or calcium channel blockers. These data, derived from a cross-sectional investigation, should be looked at as descriptive for the present patient population. Accurate information on drug induced dysglycaemia can only be derived from longitudinal (cohort) studies. In contrast, the aim of the present study was to screen for undetected dysglycaemia in the presence of other risk factors and not to outline the risk for pharmacologically induced dysglycaemia.

It has been claimed that an OGTT has a limited reproducibility in particular for establishing the presence of IGT [52]. In a study by Wallander et al. [53] patients were screened with OGTT 5 days, 3 and 12 months after an AMI. Of those who were identified to have dysglycaemia at hospital discharge 93% were still classified as such, either having diabetes (64%) or IGT (29%) after 12 months. Of patients with IGT at hospital discharge 71% hade IGT or diabetes 12 months later and 29% normal glucose metabolism while 60% of patients classified with normal glucose metabolism at discharge remained normal after 12 months and 40% had developed IGT or type 2 diabetes. Thus, an OGTT seems to be reliable enough for screening purpose. Still, a second test should be performed to confirm the diagnosis, as recommended by WHO, before the institution of treatment.

## Conclusions

Hidden dysglycaemia is very common in patients treated for hypertension and/or dyslipidaemia. To apply FINDRISC as a first step of dysglycaemia screening does not provide any benefit in such populations. Of different blood tests a single HbA1c was the least efficient due to its limited ability to detect T2DM and its inability to diagnose IGT. The combination of FPG and 2hPG was the best test for detecting T2DM and the FPG was the single best test. The latter is, however, limited by the inability to detect IGT. A pragmatic strategy, decreasing the demand for an OGTT by 21%, would be to screen all patients with FPG followed by OGTT in patients with IFG. Finding simpler methods e.g. based on a 1 hour post load glucose [54] to identify individuals at high risk of progression to diabetes and CVD in the need for more targeted prevention strategies remains an important goal.
